# Individual attractiveness preferences differentially modulate immediate and voluntary attention

**DOI:** 10.1038/s41598-023-29240-5

**Published:** 2023-02-07

**Authors:** Tom S. Roth, Iliana Samara, Juan Olvido Perea-Garcia, Mariska E. Kret

**Affiliations:** 1 Cognitive Psychology Unit, Institute of Psychology, Leiden University, The Faculty of Social and Behavioral Sciences, Wassenaarseweg 52, 2333 AK Leiden, The Netherlands; 2 Apenheul Primate Park, J.C. Wilslaan 21, 7313 HK Apeldoorn, The Netherlands; 3 Animal Behaviour & Cognition, Department of Biology, Utrecht University, Utrecht, The Netherlands; 4 Leiden Institute of Brain and Cognition (LIBC), Leiden, The Netherlands

**Keywords:** Sexual selection, Human behaviour

## Abstract

Physical attractiveness plays a crucial role in mate choice for both men and women. This is reflected in visual attention: people immediately attend towards and look longer at attractive faces, especially when they are motivated to find a partner. However, previous studies did not incorporate real-life dating decisions. Here, we aimed to combine attentional tasks with individual attractiveness ratings and a real-life mate choice context, namely a speed-dating paradigm. We investigated whether heterosexual non-committed young adults showed biases in immediate and voluntary attention towards attractive faces and preferred dating partners. In line with previous research, we found considerable individual differences in individual attractiveness preferences. Furthermore, our results showed that men had a bias towards attractive faces and preferred dating partners in the immediate attention task, while results for women were mixed. In the voluntary attention task, however, both men and women had an attentional bias towards attractive faces and preferred dating partners. Our results suggest that individual attractiveness preferences are good predictors of especially voluntary attention. We discuss these findings from an evolutionary perspective and suggest directions for future research.

## Introduction

Physical attractiveness permeates important aspects of human interaction and shapes our judgements about people. Previous research shows that people associate positive personality traits with attractive people^1,2^, consider them more cooperative^3^, and attractive people have even been shown to fare better in the labor market^4,5^. In addition, physical attractiveness has an important influence on mate choice, and its weight in shaping mate choice has important effects in fundamental aspects of our psychology, such as attention. For example, previous research has shown that people’s attention is drawn faster and for a longer duration to attractive stimuli^6,7^. However, given that human mate choice is such a fundamentally complex and multifaceted phenomenon, researchers have treated it in a wide variety of distinct approaches that may capture only some of said complexity. For example, human mate choice has been studied by focusing on cognitive processes^8–10^, attractiveness ratings^11,12^, and real-life interactions^13–15^. Even though previous studies have integrated multiple methods to investigate mate choice, no previous study has examined the influence of attractiveness on visual attention and linked this to decisions in a realistic mate choice context. Given the context-sensitivity of cognitive processes^16^, we explore how individual attractiveness preferences and partner preferences shape our immediate and voluntary attention using a novel setting. Specifically, here, we combine well-established cognitive tasks with attractiveness rating tasks and a speed-date paradigm to examine whether and how these different approaches to studying human mate choice concord.

Physical attractiveness is strongly associated with attraction to a mate^11,17^, and both women and men mention physical attractiveness as an important criterion for mate selection^18,19^. Consequently, physically attractive people have more sexual partners^20^ and a higher reproductive success^21^. From an evolutionary perspective, attractiveness has been proposed to be a cue of genetic quality in terms of health or fertility: by selecting an attractive partner, one can increase the likelihood of bearing offspring with high genetic quality^19,22^. Some studies indeed suggest that attractiveness is positively correlated with health^23,24^, although this has been heavily debated^25–27^. Accordingly, people rate attractive faces as healthier than unattractive faces^28^, although this could be the result of a general halo effect for attractive people^1,29^. Altogether, by selecting an attractive mate, humans might confer their offspring a selective advantage, thereby increasing their reproductive success.

If selecting a physically attractive mate indeed results in greater fitness, this may be reflected in specific cognitive mechanisms that help people to identify, and feel attracted to, physically attractive mates. Some of these mechanisms may be understood as perceptual biases, previously termed *sexually selective cognition*^8^. These biases have been shown to interact with different cognitive processes. For example, men and women will exert more effort to see pictures of attractive than unattractive opposite-sex stimuli^30^, although this opposite-sex bias is especially strong in men^31^. When it comes to recognition memory, people seem to specifically remember attractive faces^32,33^. Importantly, this memory bias seems to be strongest for young participants, who are at the age where they are most likely to start getting involved in romantic interactions^32^. These examples show how attractiveness can modulate human cognition.

Apart from effort and memory biases, the majority of experimental studies on cognition and mate choice have focused on processes of visual attention. Several studies show attentional biases towards physically attractive faces: they are attended to first and hold our attention for a longer time^7^. Physically attractive faces are also preferentially attended to in preferential looking paradigms^6,34^. When it comes to immediate attention, previous work has shown that people identify faces that were previously rated as attractive extremely quickly. For example, when presented with two pictures at the same time for 100 ms, participants could select the most attractive picture above chance level^35^. In addition, using a dot-probe paradigm with a slightly longer time scale of 300 ms, Roth et al.^9^ demonstrated that participants showed an attentional bias towards attractive faces paired with intermediately attractive faces, but not towards unattractive faces paired with intermediately attractive faces. However, it should be noted that attractiveness categories were predefined based on ratings by a different participant sample in this study^36^.

Such an approach is typical in studies investigating attractiveness, where traditionally researchers have focused on average ratings of general attractiveness. This approach is based on the notion that people strongly agree on which features and characteristics are attractive^37^. However, recent research has emphasized that it is important to disentangle shared and idiosyncratic contributions to judgments^38^ because ample evidence shows that beauty is—at least partly—in the eye of the beholder, as agreement on attractiveness is about 50%^39,40^. Importantly, such individual preferences can also influence date success, i.e., willingness to meet again after a first date^41^. These inter-individual variations are possibly the result of differences in environments^42^, such as culture^43^ and close social relationships^40^. Nevertheless, most traditional laboratory studies did not take idiosyncratic preferences of participants into account, even though there can be considerable inter-individual variation in judging attractiveness. Taking these individual differences into consideration might reveal more pronounced effects of attractiveness on cognition. Thus, in the present study, we aimed to examine whether and the manner in which idiosyncratic attractiveness preferences influence immediate attention.

When it comes to voluntary attention, that is, where attention is allocated when able to do so freely, multiple studies have found that participants focus their attention on their sex of interest, or on the most attractive person of their sex of interest, depending on the design. For instance, Dawson & Chivers^44,45^ presented sexually explicit stimuli to participants that contained same-sex or opposite-sex people and found that heterosexual participants fixated more on the opposite-sex stimuli. Mitrovic and colleagues^46^ extended these findings by presenting same-sex and opposite-sex stimuli varying in attractiveness to heterosexual and homosexual participants. They found that participants attended most to the attractive faces corresponding to their sexual preference. Follow-up studies modified this paradigm by using the participants' own attractiveness ratings of the stimuli, instead of predefining stimuli as attractive or unattractive, and yielded similar results: people spent more time looking at faces that they found attractive^6,34^. Thus, a plethora of studies shows that people selectively attend to the more attractive face they are presented with.

Cognition can be substantially influenced by top-down processes^16,47^, and attentional biases related to mate choice are no exception to this. More specifically, mating motivations seem to modulate attentional processing of attractiveness. For example, Ma and colleagues^48,49^ used a dot-probe paradigm with 500 ms presentation duration to study whether immediate attention was modulated by attractiveness and relationship status. They found that non-committed participants’ attention was captured by attractive faces the most, and that these same participants had trouble disengaging from attractive facial stimuli. When it comes to voluntary attention, similar results have been found: non-committed participants showed a stronger positive correlation between perceived physical attractiveness and attention than committed participants^34^. This suggests that the bias towards physical attractiveness is especially pronounced when it is adaptive, i.e., for people that might be looking for a partner.

It has been suggested that men are more attuned to physical attractiveness than women^50^. This has been supported by questionnaire studies, where women seem to place less emphasis on physical attraction of their partner than men do^51^. This is also reflected in cognition: men show a stronger correlation between stimulus attractiveness and preferential looking^34^. Similar patterns have been found in immediate attention studies^52,53^, although this finding is not always replicated^9^. However, these sex differences do not always become apparent in studies that investigate real-life interactions. On the contrary, both women and men seem to rely mostly on physical attractiveness of their partners to make mate choice decisions during speed-dates^11,13,17^. Thus, while some studies report sex differences in attractiveness bias in attentional paradigms, these differences do not seem to be reflected in dating decisions. This raises the question whether these different approaches to studying mate choice capture the same processes and to what extent they are actually informative with regard to real-life mate choice.

Here, we therefore combined two paradigms that have been used frequently to study immediate and voluntary attention in the context of human mate choice with a realistic paradigm to study human mate choice, namely speed-dating. More specifically, we investigated the association between individual preferences for attractiveness and date outcome, respectively, on immediate and voluntary attention in non-committed young adults. To test immediate attention, we employed a dot-probe task^54^. In the dot-probe task, participants briefly view two pictures presented on the display, one of which is then replaced by a dot. Participants are asked to indicate the location of the dot (right vs. left) using the corresponding keyboard keys. To investigate voluntary attention, we used a preferential looking task, where participants can freely view two stimuli in each trial^6^, while their eye movements were recorded with an eye tracker. We combined these two cognitive tasks with a speed-date paradigm in order to create a realistic mate-choice context. Speed-dating has been shown to have strong ecological validity, as participation in a speed-dating experiment can translate into real-world romantic relationships^55^. Furthermore, we aimed to examine how the results of two different but well-established types of paradigms (i.e., speed dating and cognitive tasks) relate to each other. This is because these two pervasive paradigms may be capturing fundamentally different processes relevant to mate choice that are, nonetheless, relevant to understanding the role of perceived attractiveness. As such, we believe the integration of these paradigms has the potential to more holistically inform the complex phenomenon that is human mate choice.

Our study aimed to contribute to the understanding of the interplay between cognition, attractiveness, and mate choice in two main ways. First, we linked idiosyncratic attractiveness preferences not only to voluntary, but also immediate attention. Second, we studied whether attractiveness-related attentional biases are indeed reflective of actual mate choice. Regarding our analyses, we first explored whether there were idiosyncratic differences in attractiveness ratings in our sample, as reflected in inter-rater reliability of attractiveness ratings. With regards to individual attractiveness preferences and the dot-probe task, we expected that participants would respond faster to the dot when it replaced a picture they themselves had previously rated as highly attractive; whereas they would respond slower to the dot when the distractor was a picture they had rated as highly attractive. With regards to date outcome and the dot-probe task, we expected people to respond faster to the dot when it replaced a picture of a person they later felt attracted to on a speed-date. However, we expected them to respond slower when the distractor was a picture of a person they later felt attracted to while on a speed-date. With regard to individual attractiveness preferences and preferential looking, we expected a positive association between individual attractiveness rating and looking time. Furthermore, regarding date outcome and looking time, we expected participants to look longer at people they later felt attracted to on a speed-date. For each analysis, we also explored whether the relationships would be more pronounced for men than for women.

## Method

### Participants

Eighty (*N* = 80) participants were recruited for a speed-dating event and divided into four groups of 10 male and 10 female participants. In line with the inclusion criteria, all participants reported that they were between 18 and 26 years old, heterosexual, non-committed, Dutch-speaking, and not under treatment for psychiatric disorders. All but 2 participants indicated that they were interested in pursuing a long-term relationship. Ten participants did not attend the experimental session and three participants (1 woman) withdrew their participation before the speed-dating sessions, leading to a final sample of 67 (*N* = 67; 35 women: *M*age = 22.03, *SD* = 2.26; men: *M*age = 22.61, *SD* = 1.75). All participants provided informed consent in accordance with the declaration of Helsinki. Participants received a complementary ticket to Apenheul Primate Park (Apeldoorn, the Netherlands) for their participation. The study was approved by the Leiden University Ethics Committee (CEP: 2020-02-20-M.E.Kret-V1-2169).

### Procedure

After filling in several demographic questionnaires, the researchers took profile photos of the participants against a white background and also collected auditory and olfactory material, which will not be described in this paper. Hereafter, all participants completed a battery of cognitive tasks (the full methods are described in the Supplemental Material; preregistered using the AsPredicted database 36,394). Here, we focus on the dot-probe, preferential looking task, and attractiveness rating task. All tasks were controlled by an E-prime script (E-prime version 3; Psychology Software Tools, Pittsburgh, PA) in conjunction with the E-Prime Extensions for Tobii Pro (EET) for the preferential looking task. All stimuli were presented against a gray background. Furthermore, all tasks were presented on an 23.8-inch HP EliteDisplay 243 m monitor with 1680 × 1050 resolution and 60 Hz refresh rate.

In the dot-probe task, participants briefly view two pictures of the presented on the display, one of which is then replaced by a dot. Participants are asked to indicate the location of the dot (right vs. left) using the corresponding keyboard keys. In our study, all stimuli consisted of the opposite-sex participants' profile photos from the same group. In the case that one group consisted of fewer than 10 individuals, pictures of opposite-sex participants from the previous group were added to keep the number of trials consistent across participants. It is important to note that participants had not met their partners at that point in the experimental procedure and thus could not have known that these were replacement pictures. Each trial started with a centrally presented fixation cross for a jittered duration between 1020 and 1260 in increments of 60 ms. Next, participants viewed the pictures of two opposite-sex participants for 300 ms, one of which was then replaced by a dot until the participant indicated the correct location using the corresponding keyboard keys (*z* for left, *m* for right). Every trial ended with an inter-trial interval between 1380 and 1620 ms in increments of 60 ms. After completing 10 practice trials, participants viewed all possible combinations of the opposite-sex participants' photos (i.e., 45 dyads) twice, so each participant in a dyad would be presented as the probe (i.e., the picture replaced by the dot) and the distractor picture (i.e., the picture not replaced by the dot) leading to a total of 100 trials. Location of the probe and distractor pictures was pseudo-randomized across the trials. The task lasted approximately 8 min.

In the preferential looking task, in each trial, participants viewed two of the opposite-sex participants' pictures while their eye movements were recorded using an X2-60 Tobii eye-tracker (Tobii Pro, 2014) at a sampling rate of 60 Hz. Participants placed their chin on a chin rest at approximately 50 cm from the monitor. Each trial started with a centrally presented fixation cross for 720 ms, followed by the two pictures presented on the display for 3000 ms. Similar to the dot-probe task, in the case that one group consisted of fewer than 10 individuals, pictures of opposite-sex participants from the previous group were added to keep the number of trials consistent across participants. Every trial ended with a jittered ITI between 1380-1620 ms in increments of 60 ms. After performing 3 practice trials, participants completed 45 trials. The task lasted approximately 6 min.

In addition to the tasks described above, participants rated the attractiveness of all of the stimuli on a 7-point scale. The stimuli were presented sequentially for 3 s on a computer monitor, after which the participants could indicate how attractive they found the person in the stimulus. The order of the tasks was randomized between participants.

After completing the tasks, participants went on a maximum of ten 4-min speed-dates^14,56^. Men and women were seated at opposite sides of a table, their view of their partner occluded by a barrier. At the start of each date, the barrier was removed, and following the ring of the bell, participants had a four-minute date with their partner. After 4 min, participants indicated the date outcome, i.e., whether they would be interested in going on another date with them (yes/no); their prediction about whether their partner would be interested to go on another date with them (yes/no); and whether they knew their partner before the date (yes/no). Furthermore, we asked participants to indicate how attractive they found their partner (7-point scale) and how attractive they considered them as a long-term mate (7-point scale). It should be noted that these questions referred to attractiveness in general, and not specifically physical attractiveness. Participants had one minute to fill in the questionnaire after each date. Next, male participants rotated to their next prospective partner. After completing all possible date combinations, participants were debriefed about the purposes of the study.

### Data processing

#### Dot-probe

In total, 58 participants completed the dot-probe task. In the second female group, we could not collect dot-probe data due to a technical issue. In total, we had 5220 datapoints for the dot-probe task before data filtering. One participant did not complete the pre-date attractiveness rating task. Therefore, we excluded this participant’s data (90 trials) from the analysis that investigated the effect of attractiveness on immediate attention, leaving us with data from 57 participants. Next, we excluded outliers by subject: as a lower boundary, we used 200 ms for anticipatory reaction times^57^. We calculated the upper limit by subject following Leys and colleagues ^58^: we calculated the median absolute deviation (MAD) per subject and the median RT per subject. We then used a moderately conservative criterion to exclude trials: if the RT was slower than the subject’s median RT + 2.5 * MAD, we excluded the trial. These outlier criteria resulted in the exclusion of 299 trials (5.83%). Hereafter, we centralized the RTs by subject. This was done to make it easier to set a prior for the Intercept. All factorial predictors were sum coded, and pre-date attractiveness ratings were centered at 4 because this was the middle option.

We followed a similar procedure for the analysis that investigated the association between date outcome (i.e., willingness to go on another date with dating partner) and post-date attractiveness rating on immediate attention. Two participants dropped out before the speed-date part of the experiment. Therefore, we had to exclude their data, leaving us with data from 56 participants. Some participants did not go on a speed-date with every opposite-sex person they saw on the stimuli, either due to dropouts or unequal group size. After excluding the cases where date outcomes were missing for either the probe or the distractor stimulus, we ended up with 3460 data points out of the original 5220. Hereafter, we again excluded outliers by subject (see above), resulting in the exclusion of 209 trials (6.04%).

#### Eye-tracking

In total, 36 participants completed the eye-tracking task. One participant did not complete the pre-date attractiveness rating task and did not participate in the speed-dates. Therefore, we excluded their data (45 trials) from the analysis. Furthermore, we excluded 6 trials because participants were not looking at the stimuli, leaving us with 1569 trials from 35 participants to investigate the effect of attractiveness on voluntary attention. For the analysis that investigated the effect of date outcome and post-date attractiveness rating on voluntary attention, we had a smaller number of trials due to the fact that not all people that were rated for attractiveness participated in the speed-dates (either due to dropout or due to unequal group sizes). In total, we could include 1009 trials from 35 participants.

Eye-tracking data were recorded continuously throughout the task with a sampling rate of 60 Hz. Here, only data during the stimulus presentation were analyzed. Fixations on either area of interest (AOI) were logged using a custom E-prime script. We excluded practice trials (6.25%) and gaze samples where either the left or right pupil was not recorded (3.50%). Following these criteria, we were left with 90.25% of the data intact.

### Statistical analyses

All analyses were performed in R statistics Version 4.1.3^59^. First, we calculated the Intra-Class Correlations (ICC) for the individual pre-date attractiveness ratings. We used the R package *irrNA*^60^, because it properly deals with missing values in the computation of ICC. In line with recommendations from McGraw & Wong^61^ we used the ICC(A, 1) to test for absolute agreement between rates. We report the ICC estimate and the 95% confidence interval.

Furthermore, we used the R package *correlation*^62^ to test the relationship between pre-date attractiveness ratings, post-date attractiveness ratings, and date outcome. The *correlation* package allows for computation of a wide variety of correlations, such as Bayesian multilevel correlations. In our case, we used Bayesian multilevel Spearman correlations to investigate the association between pre-date and post-date attractiveness ratings. To test the relationships between date outcome and pre-date and post-date attractiveness ratings, respectively, we used Bayesian point-biserial correlations. These analyses were based on a dataset that consisted of only complete cases for all three variables of interest. In total, this concerned 482 datapoints of 58 participants.

For our main analyses, we used Bayesian mixed models. Bayesian analyses have gained in popularity over the past few years because they offer a number of benefits compared to frequentist analyses^63,64^. While frequentist methods (e.g., *p*-value null-hypothesis testing^65^) inform us about the credibility of the data given a hypothesis, Bayesian methods inform us about the credibility of our parameter values given the data that we observed. This is reflected in the different interpretation of frequentist and Bayesian confidence intervals: The first is a range of values that contains the estimate in the long run, while the latter tells which parameter values are most credible based on the data^63,66^. Furthermore, Bayesian methods allow for the inclusion of prior expectations in the model, are less prone to Type I errors, and are more robust in small and noisy samples^64^. Altogether, these reasons make Bayesian methods a useful tool for data analysis.

All models were created in the Stan computational framework and accessed using the *brms* package^67,68^, version 2.17.0. All models were run with 4 chains and 5000 iterations, of which 1000 were warmup iterations. We checked model convergence by inspecting the trace plots, histograms of the posteriors, Gelman-Rubin diagnostics, and autocorrelation between iterations^69^. We found no divergences or excessive autocorrelation in any model.

#### Dot-probe

To analyze the dot-probe data we used Bayesian mixed models with a Gaussian distribution. First, to study the association between attractiveness and immediate attention, we modeled *Reaction time* (mean-centered by subject) as a function of *Attractiveness rating of probe picture* and *Attractiveness rating of distractor picture*, and their interactions with *Gender*. We allowed the intercept and the effects of *Attractiveness rating of probe picture* and *Attractiveness rating of distractor picture* to vary by *Subject.* Second, to study the association between date outcome (i.e., willingness to go on another date with dating partner) and immediate attention, we followed the same procedure as described above. However, the predictors *Attractiveness rating of probe picture* and *Attractiveness rating of distractor picture* were replaced with *Date again probe picture* (binary: yes/no) and *Date again distractor picture* (binary: yes/no), also in the random effect formula.

We used a Gaussian prior with *M* = 0 and *SD* = 2.5 for the Intercept of the model. For the independent variables, we specified regularizing Gaussian priors with *M* = 0 and *SD* = 5. For all variance parameters, we kept the default Student’s *t* priors with 3 degrees of freedom. After running the models, we used the *emmeans*-package^70^ to obtain estimates and pairwise contrasts based on the posterior predictive distribution. Using these values, we calculated multiple quantitative measures to describe the effects. First, we report the median estimate *b,* and median absolute deviation of the estimate between square brackets. Second, we report an 89% credible interval of the estimate (89% CrI). We have chosen 89% instead of the conventional 95% to reduce the likelihood that the credible intervals are interpreted as strict hypothesis tests^66^. Instead, the main goal of the credible intervals is to communicate the shape of the posterior distributions. Third, we report the probability of direction (*pd*), i.e., the probability of a parameter being strictly positive or negative, which varies between 0.5 and 1^64^.

#### Eye-tracking

To analyze the eye-tracking data, we used a zero–one inflated beta model, which is suitable for continuous proportions containing zeros and ones. These models consist of two components, namely a beta component to describe the values between 0 and 1, and a binary component to predict the occurrences of zeros and ones^71^. For each trial we calculated a *Looking time bias score* by dividing the time fixating on the left picture by the total time fixating on the pictures. Thus, this score reflects the proportion of fixation time spent looking at the left picture. In looking time studies, it is common practice to calculate a looking time bias (proportion of total looking time). In the case of clear categories, this is no problem. For example, imagine a study where one examines attention to attractive vs. unattractive faces. One could calculate a looking time bias by calculating the proportion of time looking at the attractive face for all trials. However, in our case, we have no categorical variables but continuous ones, namely attractiveness ratings. Thus, we cannot calculate an informative bias like in the example above. Therefore, we have used the location of the photos as a reference point to calculate the looking time bias, by calculating the bias toward the left picture. Hereafter, we have tested whether this bias is affected by (1) the attractiveness ratings of the left and right picture, and (2) date outcome.

To study the association between attractiveness and voluntary attention, we modelled *Looking time bias score* (proportion of time looking at the left picture) as a function of *Attractiveness rating of the left picture* and *Attractiveness rating of the right picture*, and their interactions with *Gender*. We allowed the intercept to vary by *Subject*. Importantly, we weighed each trial by the looking time in that trial relative to the subject’s average (see Data Processing). Thus, trials in which the participant paid more attention to the screen had a larger weight in the analysis. In this manner, we avoided that trials where participants were distracted or disinterested would have a large influence on the outcome of our analysis. Furthermore, we specified the same formulas for the precision parameter (*phi*; shape of the beta distribution), the zero–one inflation parameter (*zoi*; probability of observing a zero or a one), and the conditional one-inflation parameter (*coi*; probability of observing a one if a zero or one is observed). To study the association between date outcome (i.e., willingness to go on another date with dating partner) and voluntary attention, we followed the same procedure as described above. However, the predictors *Attractiveness rating of the right picture* and *Attractiveness rating of the left picture* were replaced with *Date again right picture* (binary: yes/no) and *Date again left picture* (binary: yes/no).

We used a Gaussian prior with *M* = 0 and *SD* = 0.25 for the Intercept of the beta component of the model. For the independent variables, we specified regularizing Gaussian priors with *M* = 0 and *SD* = 0.5. This also applied to the independent variables in the formulas for *phi, coi,* and *zoi*. For all variance parameters, we kept the default Student’s *t* priors with 3 degrees of freedom. Furthermore, we kept the default logistic priors for the Intercepts of *zoi* and *coi*, and default Student’s *t* prior with 3 degrees of freedom for the Intercept of *phi*.

After running the models, we used the *emmeans*-package^70^ to integrate the different model components, and provide estimates based on the posterior predictive distribution. Using these values, we calculated multiple quantitative measures to describe the effects (see Statistical analyses; Dot-probe). It is important to note, though, that the predictions are on the response scale (probability). This complicates interpretation for the continuous variables, because the slope on the response scale is not constant but is shallower or steeper depending on the value of the continuous variable. In the text we report the effect size measures for when the continuous variable of interest is set at 0, but in the Supplementary Material we provide similar measures for other values of the continuous variable of interest.

#### Model comparisons

For both the dot-probe and eye-tracking analyses, we additionally created a complete cases dataset in which we included only those cases for which we had pre-date attractiveness ratings, post-date attractiveness ratings, and date outcomes. Using these two datasets, we again ran the analyses described above (with pre-date attractiveness, post-date attractiveness, or date outcome as predictor, respectively). Hereafter, we used leave-one-out cross validation (PSIS-LOO-CV^72^) to calculate the expected log predictive density (elpd_LOO_), which quantifies predictive accuracy for each model. Then, we calculated the difference in elpd_LOO_ (Δelpd_LOO_) between all three models. If Δelpd_LOO_ of two models is at least two SEs, this suggests that the models substantially differ in predictive performance^73^. Therefore, we report both the Δelpd_LOO_ and the SE of the difference. In total, the immediate attention dataset consisted of 3198 trials of 55 participants, while the voluntary attention dataset consisted of 1009 trials of 35 participants.

## Results

### Inter-rater agreement on attractiveness

When examining the inter-rater agreement on pre-date attractiveness ratings, we found an ICC(1, A) of 0.42 (95% CI [0.32; 0.52]). This result suggests that participants differed in their attractiveness preferences independent of gender. Furthermore, we explored the inter-rater agreement for men and women separately. For women, we found an ICC(1, A) of 0.25 (95% CI [0.14; 0.41]), while for men, we found an ICC(1, A) of 0.50 (95% CI [0.39; 0.64]). These results suggest that women had substantially lower agreement than men.

### Correlations between attractiveness ratings and date outcome

We found that pre-date attractiveness rating, post-date attractiveness rating, and date outcome all showed a strong correlation. First, a point-biserial correlation indicated that pre-date attractiveness rating and date outcome were correlated (*r* = 0.44, 89% CrI [0.36; 0.50], *pd*_+_ = 1.00). Second, we found that post-date attractiveness showed an even stronger correlation with date outcome (*r* = 0.67, 89% CrI [0.62; 0.71], *pd*_+_ = 1.00). Third, a Spearman correlation showed that pre-date and post-date attractiveness were correlated (*r* = 0.57, 89% CrI [0.51; 0.62], *pd*_+_ = 1.00).

### Immediate attention (dot-probe)

#### Pre-date attractiveness ratings

**Figure 1:**
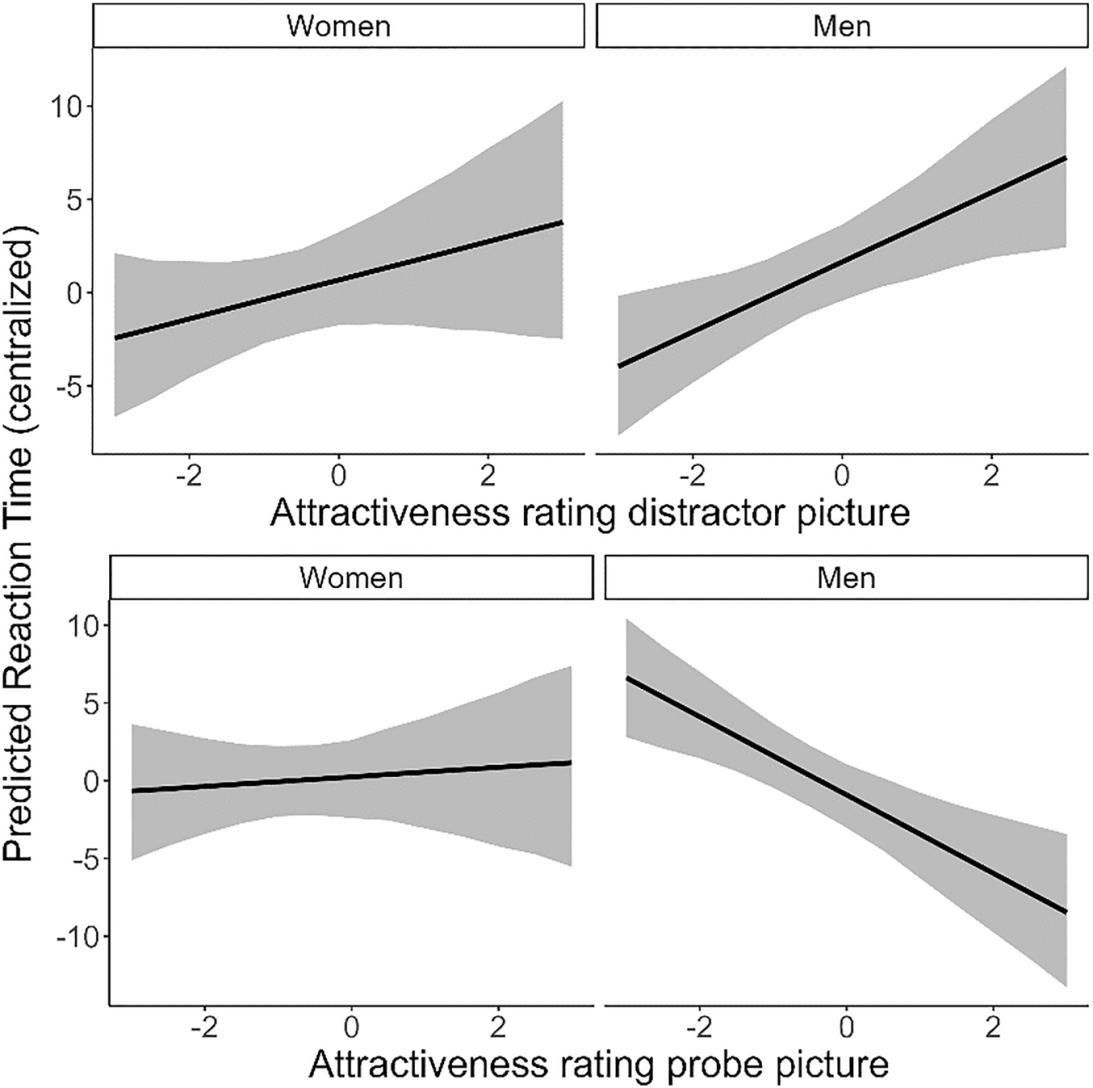
Conditional effect plot showing associations between *Pre-date attractiveness rating* and *Reaction Time* (RT) separate per *Gender*. The black line represents the median effect, while the grey ribbon represents the 95% credible interval.

We first examined the association between *Pre-date attractiveness rating* and *Reaction time* using a Bayesian mixed model with Gaussian distribution (Descriptives: Table S1-3; Model Table: Table S4). We found a robust overall effect of *Pre-date attractiveness rating of distractor picture* on *Reaction time* (*b* = 1.46 [0.53], 89% CrI [0.60; 2.29], *pd*_+_ = 1.00), with participants responding slower by 1.46 ms to the probe when there is an increase of 1 in attractiveness ratings of the distractor picture. There was no robust interaction with *Gender* (*b*_women-men_ = − 0.83 [1.06], 89% CrI [ − 2.49; 0.88], *pd*_-_ = 0.79). However, after visually inspecting the results, we wanted to explore whether the positive effect of *Pre-date attractiveness rating of distractor picture* on *Reaction Time* was robust within each level of *Gender.* We found that the effect was indeed robust for men (*b*_men_ = 1.87 [0.65], 89% CrI [0.80; 2.88], *pd*_+_ = 1.00), but not for women (*b*_women_ = 1.04 [0.83], 89% CrI [ − 0.33; 2.34], *pd*_+_ = 0.89). Thus, men responded slower to the probe by 1.87 ms when the attractiveness rating of the distractor picture was increased by 1, while no robust effect was found for women (see Fig. 1 top panel).

Furthermore, we found a robust overall effect of *Pre-date attractiveness rating of probe picture* on *Reaction time* (*b* = − 1.11 [0.55], 89% CrI [− 1.97; − 0.24], *pd*_-_ = 0.98), whereby participants responded faster by 1.11 ms when the attractiveness rating for the probe picture was increased by 1. In this case, however, the effect was modulated by *Gender* (*b*_women-men_ = 2.83 [1.06], 89% CrI [1.12; 4.51], *pd*_+_ = 1.00)*.* Therefore, we further explored the slope per *Gender.* We found a robust negative effect of *Pre-date attractiveness rating of probe picture* for men (*b*_men_ = − 2.51 [0.67], 89% CrI [− 3.59; − 1.48], *pd*_-_ = 1.00), indicating that men responded faster by 2.51 ms when the attractiveness rating of the probe picture was increased by 1. For women, on the other hand, we found no robust effect (*b*_women_ = 0.30 [0.85], 89% CrI [− 1.03; 1.59], *pd*_+_ = 0.64). Thus, men seemed to respond faster to the probe when they considered the image that was replaced by the probe highly attractive, while no robust effect was found for women (see Fig. 1 lower panel).

We performed the same analysis with the *Post-date attractiveness ratings* as predictor. This analysis yielded the same results (Table S5).

#### Date outcome

**Figure 2:**
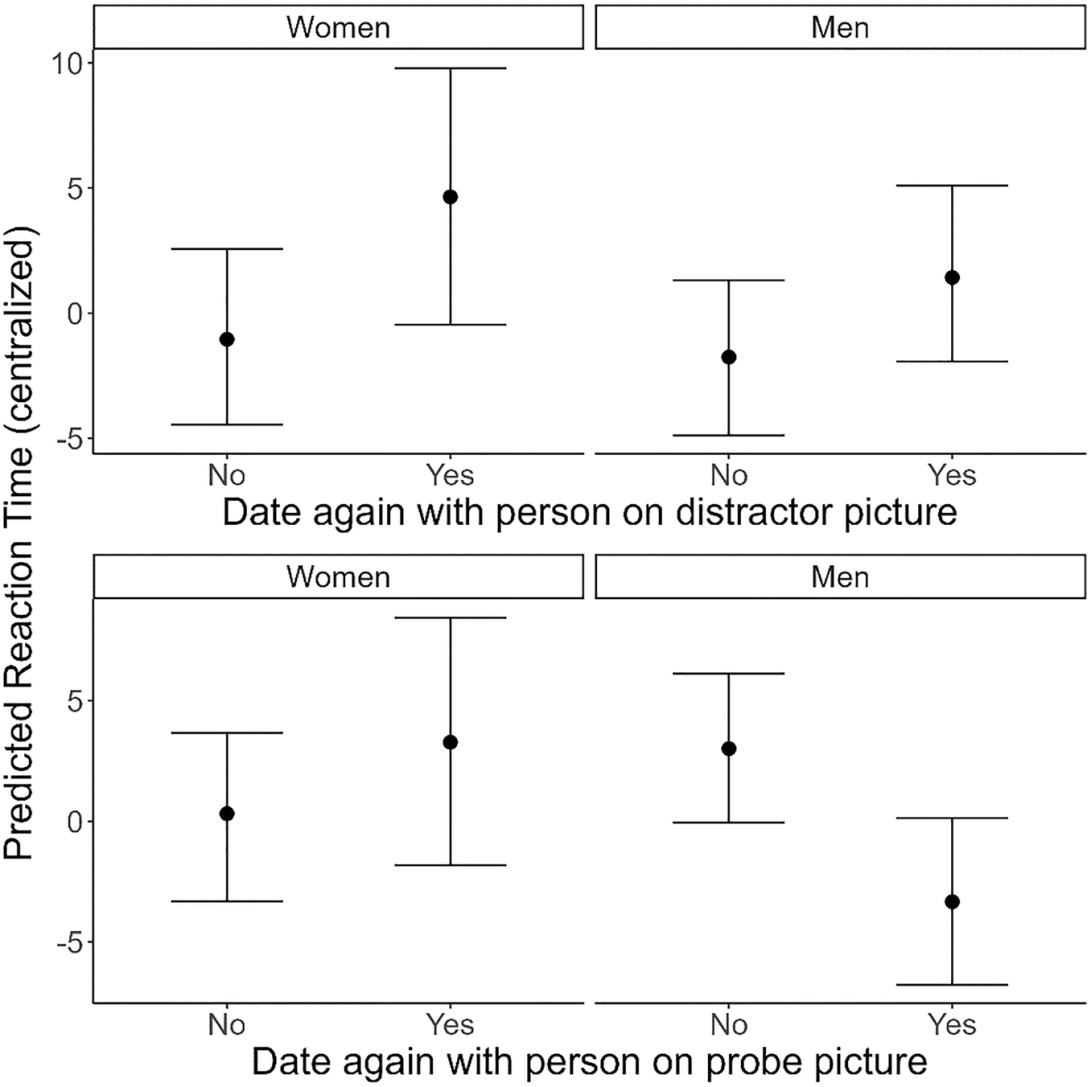
Conditional effect plot showing the effect of *Date outcome* on *Reaction Time* (RT) separate per *Gender*. Values are conditioned on the other predictor set to “No”. Error bars represent 95% Credible Intervals.

Second, we investigated the association between *Date outcome* and *Reaction Time* using a Bayesian mixed model with a Gaussian distribution (Descriptives: Table S6-8; Model Table: Table S9). We found a robust effect of *Date again distractor picture* on *Reaction time*: participants were slower by 4.41 ms to respond to the probe if the distractor image depicted someone they later indicated as a successful date compared to when the distractor image depicted someone that they did not consider a successful date during their speed-dates (*b*_no-yes_ = − 4.41 [1.96], 89% CrI [− 7.51; − 1.29], *pd*_-_ = 0.99), and this effect did not substantially differ per *Gender* (*b*_women-men_ = − 2.48 [3.93], 89% CrI [− 10.30; 5.26], *pd*_-_ = 0.74; see Fig. 2 top panel).

When investigating the effect of *Date again probe picture* on *Reaction Time*, we did not find a robust overall effect (*b*_no-yes_ = 1.68 [1.94], 89% CrI [− 1.49; 4.70], *pd*_+_ = 0.81). However, we did find a robust interaction with *Gender* (*b*_women-men_ = − 9.33 [3.88], 89% CrI [− 16.80; − 1.60], *pd*_-_ = 0.99). Therefore, we explored the effect of *Date again probe picture* within each level of *Gender.* For women, we found no robust effect (*b*_women_ = − 2.97 [3.06], 89% CrI [− 8.96; 3.12], *pd*_-_ = 0.84). For men, on the other hand, we found that they responded faster to the probe by 6.33 ms when it replaced a picture of someone whom they later considered a successful date during their speed-dates (*b*_men_ = 6.33 [2.36], 89% CrI [1.75; 11.00], *pd*_+_ = 1.00; see Fig. 2 lower panel).

### Voluntary attention (eye-tracking)

#### Pre-date attractiveness ratings

We first explored the association between *Pre-date attractiveness rating* and *Looking time bias*, using Bayesian zero–one inflated beta regression (Descriptives: Table S10-12; Model Table: Table S13). We found that attractiveness ratings had a robust effect on voluntary attention. More specifically, participant’s attractiveness ratings of the left picture correlated positively with proportion of time spent looking at the left picture (*b* = 0.087 [0.0050], 89% CrI [0.079; 0.095], *pd*_+_ = 1.00), while we found the opposite effect for the attractiveness rating of the right picture (*b* = − 0.098 [0.0041], 89% CrI [− 0.106; − 0.091], *pd*_*-*_ = 1.00). The results were similar for other values of *Pre-date attractiveness rating*: increased attractiveness ratings of the left picture were associated with an increased probability of looking at the left picture, while the opposite was true for the right picture (Table S14).

To see whether the effect was modulated by *Gender*, we compared the slopes for men and women. However, we found no robust interaction between *Gender* and *Pre-date attractiveness rating* for both the left picture (*b*_women-men_ = − 0.001 [0.010], 89% CrI [− 0.026; 0.007], *pd*_-_ = 0.83) and the right picture (*b*_women-men_ = 0.012 [0.010], 89% CrI [− 0.006; 0.032], *pd*_+_ = 0.91). This pattern was similar for other values of *Pre-date attractiveness rating*: there was no robust difference in slope between men and women (Table S15).

**Figure 3:**
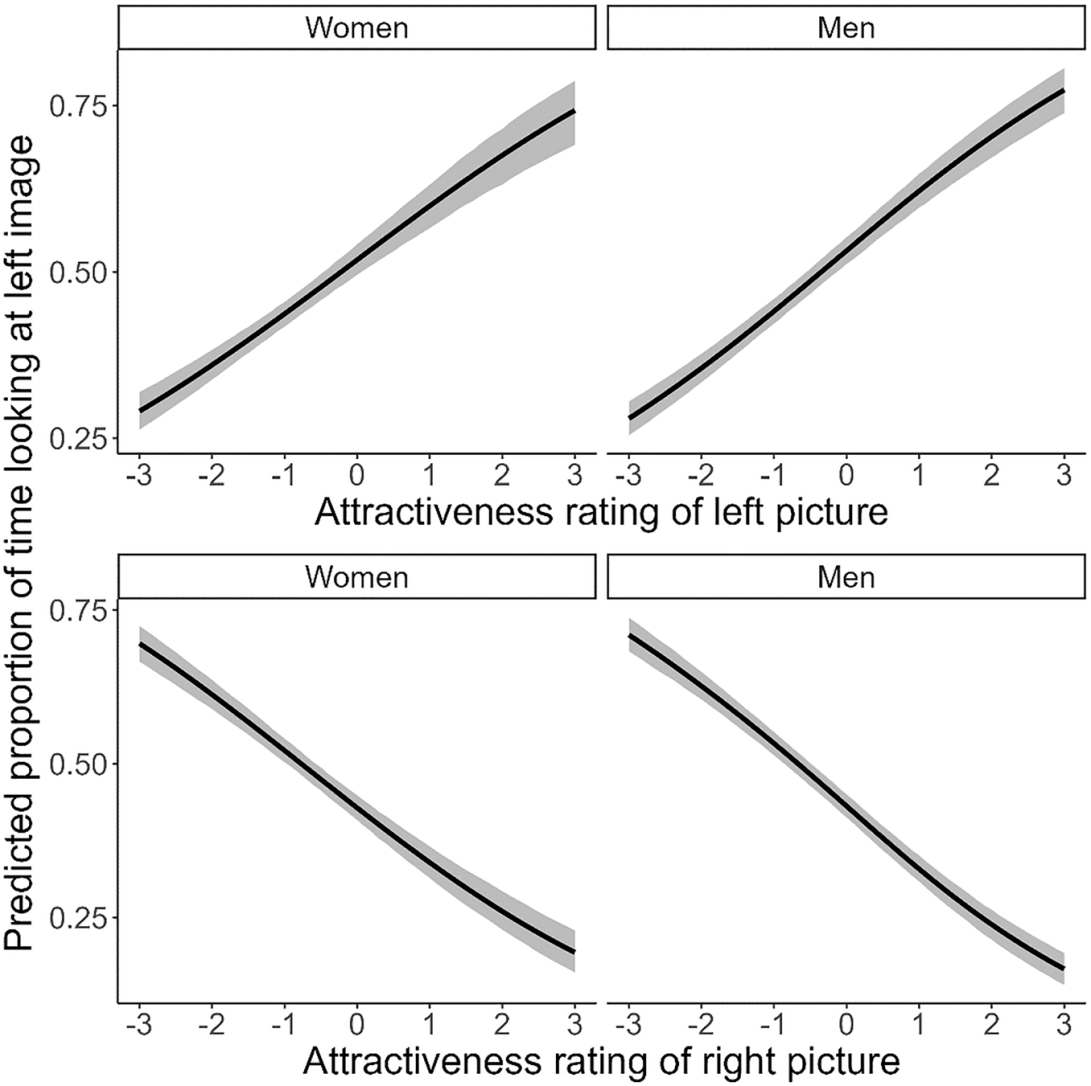
Effect plot showing associations between *Pre-date attractiveness rating* and *Looking time bias* separate per *Gender*. The black line represents the median effect, while the grey ribbon represents the 95% credible interval.

We performed the same analysis with the *Post-date attractiveness ratings* as predictor. This analysis yielded the same results (Table S16). Altogether, the results show that participants indeed looked longer at faces that they rated as attractive. The results are visualized in Fig. 3.

#### Date outcome

Second, we investigated the association between *Date outcome* and *Looking time bias* using Bayesian zero–one inflated beta regression (Descriptives: Table S17-19; Model Table: Table S20). We found that participants showed more attention towards pictures of people that they later indicated they would like to date again. More specifically, when the left picture depicted someone they wanted to date again, they spent on average 12.4 percentage points longer looking at the left picture than when the left picture depicted someone they did not want to date again (*b*_no-yes_ = − 0.124 [0.019], 89% CrI [− 0.154; − 0.095], *pd*_-_ = 1.00). When the right picture depicted someone they wanted to date again, they spent on average 15.8 percentage points less looking at the left picture than when the right picture depicted someone they did not want to date again (*b*_no-yes_ = 0.158 [0.017], 89% CrI [0.131; 0.186], *pd*_+_ = 1.00).

To see whether the effect was modulated by *Gender*, we investigated whether the effect for women and men was substantially different. However, we found no consistent gender differences (Left picture: *b*_women-men_ = 0.060 [0.037], 89% CrI [0.002; 0.118], *pd*_+_ = 0.95; Right picture: *b*_women-men_ = 0.014 [0.034], 89% CrI [− 0.043; 0.066], *pd*_+_ = 0.66), although the *pd* suggested that the effect of *Date outcome* on *Looking time bias* was stronger for men for the left picture specifically.

**Figure 4:**
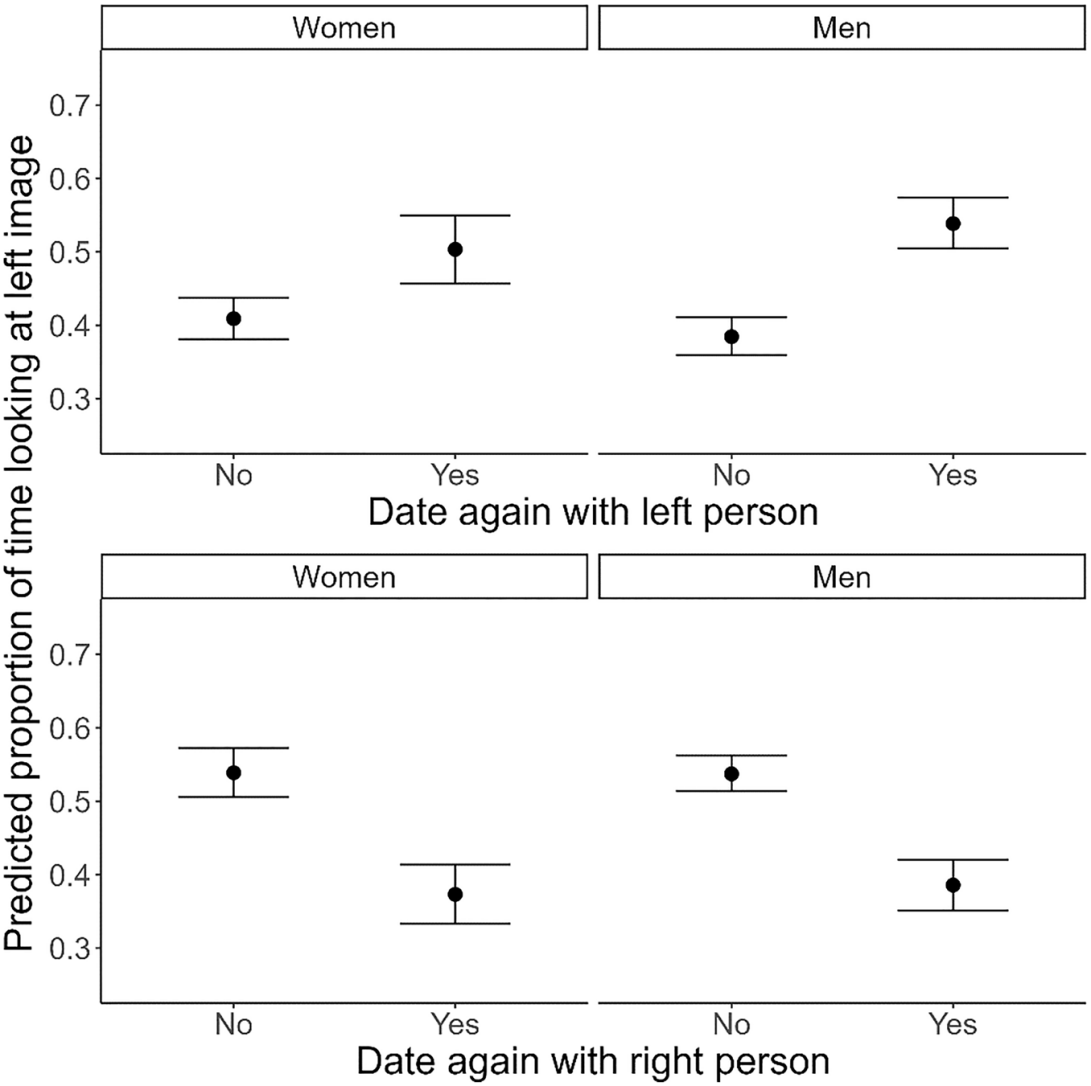
Plot showing the effect of *Date outcome* on *Looking time bias* separate per *Gender*. Error bars represent 95% Credible Intervals.

Altogether, the results show that participants indeed looked longer at the faces of people that they later indicated they wanted to see again after their speed-date (Fig. 4).

### Model comparisons

Regarding the immediate attention analysis, we found no clear differences in predictive accuracy between the three models (Table S21-23). Although the model that included *Pre-date attractiveness rating* had the highest expected log-predictive density, the differences with the models that included *Post-date attractiveness rating* (Δelpd_LOO_ = 10.5 [9.6]) or *Date outcome* (Δelpd_LOO_ = 14.0 [11.2]) as predictors were not robust due to the relatively high standard errors. Thus, while the model that incorporated *Pre-date attractiveness rating* as predictors had the highest predictive accuracy, there was no substantial difference in predictive accuracy with the two other models.

Regarding the voluntary attention analysis, we found robust differences in predictive accuracy between the three models (Table S24-26). Namely, the model that included *Pre-date attractiveness rating* had a substantially higher predictive accuracy than the models included *Post-date attractiveness rating* (Δelpd_LOO_ = 100.1 [17.4]) or *Date outcome* (Δelpd_LOO_ = 133.5 [17.9]) as predictors. Thus, the model comparisons suggested that pre-date attractiveness ratings might be a better predictor of voluntary attention than post-date attractiveness ratings or date outcome.

## Discussion

Here we combined a naturalistic speed-date paradigm with cognitive tasks on attentional biases to investigate how physical attractiveness shaped processes of immediate and voluntary attention, using a dot-probe task and a preferential looking paradigm, respectively. First, consistent with previous literature, we found considerable variation in attractiveness ratings between subjects. With regard to immediate attention, we found that only men’s attention was modulated by attractiveness, but we found no consistent association between date outcome and immediate attention. With regard to voluntary attention, we found that both men and women looked longer at faces that they rated as attractive before their date. Furthermore, participants showed more attention towards the faces of people that they later indicated they wanted to date, suggesting that voluntary attention can to some extent reflect mate choice for both men and women. However, model comparisons showed that pre-date attractiveness ratings were more predictive of immediate and voluntary attention than date outcome and post-date attractiveness, although the results are not unequivocal for immediate attention. Below, we discuss these findings and address possible implications and limitations of our study.

Similar to previous work on (dis)agreements in attractiveness ratings, we found an ICC of approximately 0.4 for the pre-date attractiveness ratings^39,40^, reinforcing the idea that individual attractiveness preferences can vary and should be taken into account when studying cognitive aspects of mate choice. Consistent with this idea, we found that individual attractiveness ratings predicted immediate attention in men. This finding extends previous work^9^ on immediate attention and attractiveness that showed a general attentional bias for faces that were predefined as attractive in a large community sample, but found no effect of sex on this bias. Crucially, this previous study did not account for relationship status. Given that motivation can influence immediate attention^48,49,52^, we only tested non-committed participants who were interested in a relationship, and as mentioned above we used their individual attractiveness ratings as predictor instead of pre-defined categories. Thus, we can conclude that men immediately attended towards faces that they rated as attractive, but we did not find the same result for women. Future research should aim to disentangle and quantify the effects of general attractiveness and individual attractiveness preferences on attention. For example, a recent study on dating behavior^41^ showed that both general and individual preferences uniquely contribute to date outcome, but whether this is also the case for attentional processes remains unknown.

It is tempting to interpret our results on immediate attention as evidence for the notion that men are more attuned towards attractiveness than women, which has also been found in previous immediate attention studies^52,53^. However, in our exploratory analysis of the interaction between pre-date attractiveness rating and gender, we found a robust gender difference only for the effect of probe picture attractiveness on reaction time. While the effect of distractor picture attractiveness was robust only for men, the difference between men and women itself was not robust. Therefore, we refrain from interpreting the differences between men and women as clear evidence for a sex effect, as previous studies have described the pitfalls of interpreting differences in post-hoc effects as evidence for a robust interaction^74,75^. With regard to the absence of a robust effect in women, in accordance with our findings, previous work has shown that the neural activity of men and women might differ in response to faces varying in attractiveness. Van Hooff and colleagues^76^ investigated the neural underpinnings of processing attractiveness. They found higher late positive event-related potential (ERP) amplitudes (250–600 ms post cue) in men than women. Crucially, this ERP has been linked to appraisal of facial attractiveness^77^. This finding suggests that men might appraise attractiveness differently than women, which could translate into observable differences in processes involving immediate attention^76^. Future research should further investigate the neural underpinnings of appraising attractiveness and how these translate to behavior.

Previous studies on immediate attention and attractiveness heavily relied on consensus attractiveness ratings^9,48,49,52^. Here, we examined whether taking the idiosyncratic preferences into account rather than general attractiveness ratings would increase the magnitude of the effect sizes found in the dot-probe task as compared to previous literature. We found that people in general responded 7 ms faster between the least and highest attractiveness rating. However, the difference between the two most extreme conditions (a very unattractive probe picture paired with a very attractive distractor picture, and the other way around) would be ~ 15 ms. This effect size is similar to those that have been typically reported in dot-probe studies^78^. Regarding the effect of attractiveness on immediate attention, this effect is comparable to a previous study that did not take idiosyncratic preferences into account^9^. In that study, people had an attentional bias of ~ 9 ms to attractive faces when paired with neutral faces, but had a ~ 6 ms attentional bias to neutral faces when these were paired with unattractive faces. Overall, this indirectly translates to a ~ 15 ms attentional bias to attractive faces compared with unattractive faces. While it is important to note that this is an indirect comparison, and that the methods are slightly different, this effect size fits well with our current finding. In conclusion, contrary to our expectation, taking idiosyncratic preferences into account did not increase the magnitude of previously recorded effects of attractiveness on immediate attention. Instead, the size of the effect of consensus ratings and idiosyncratic ratings on immediate attention seem to be rather similar.

Our hypothesis regarding date outcome and immediate attention were partly supported. Specifically, we found an overall effect of the distractor picture on RT, and an effect of the probe picture for men but not for women. For men, these results are in line with our previously described effects of attractiveness on immediate attention. Given that we found a robust association between attractiveness and immediate attention for men, and that we know that date outcome is strongly associated with attractiveness^11,17^, it is not surprising that date outcome and immediate attention are associated as well. Of course, physical attractiveness ratings do not perfectly predict date outcome; other processes such as physiological linkage^15^, nonverbal behavior^79^, attachment styles^80^ and perceived similarity^81^ all explain date outcome to some extent as well. Still, the association between attractiveness rating and date outcome might have been strong enough to explain the association between date outcome and RT in the immediate attention task.

In the preferential looking task, we found that both men and women divided their attention based on the attractiveness of the stimuli they were presented with. This is in line with previous work^6^, but also contrasts with other work that found a gender difference, with men showing a stronger association between voluntary attention and attractiveness than women^34^. However, it is important to note that participants in our study were all interested in a relationship, i.e., they were motivated to find a partner, while other studies tested both non-committed and committed participants^34^. As has been suggested, motives can substantially affect cognitive processes^16^. On top of that, participants in our study were aware that they would later meet the people they saw during the tasks, possibly strengthening their motivation even further.

The preferential looking task consisted of trials with a prolonged exposure to the stimuli compared to the dot-probe task. Therefore, participants were able to freely look upon the stimuli and gather more relevant information from the stimuli compared to the dot-probe task. Given that women might need more contextual information in order to appraise a potential partner^82^, this could possibly also explain why we do not find any sex differences in the preferential looking task, while we do find some evidence for sex differences in the dot-probe task. Future research should further investigate the concordance between immediate and voluntary attention to attractiveness and their relationship to gender.

We also found that date outcome was substantially associated with voluntary attention: participants indicated that they wanted to date again with people that they looked at for longer during the preferential looking task. This again highlights the strong association between attractiveness ratings and initial partner preferences: especially on first dates people seem to employ physical attractiveness as their main selection criterion^11,17^. Given the strong association between attractiveness rating and voluntary attention, it is not surprising that the association between date outcome and voluntary attention is also robust. An exploratory analysis showed that the associations were not modulated by gender: both men and women showed highly similar trajectories with regard to attractiveness-contingent voluntary attention. Importantly, we consider it unlikely that this effect is driven by uncertainty in the parameter estimates, given that the credible intervals for the interactions between attractiveness rating and gender were very narrow (see Fig. 3). Despite the fact that this finding is somewhat inconsistent with evolutionary theories of human mate choice that emphasize sex differences in attractiveness appraisal^18,83^, it is in line with previous speed-dating studies that failed to find gender differences in the appreciation of physical attractiveness^11,13,17^. Here, we have extended these findings by showing that both individual attractiveness ratings and date outcome are associated with voluntary visual attention in both men and women. Nonetheless, it should be noted that these analyses were exploratory in nature, and thus no strict inference can be drawn.

One could argue that it is not readily clear whether our findings (both in immediate and voluntary attention tasks) reflect long-term or short-term mate choice dynamics. Previous studies have questioned the ecological validity of speed-date paradigms to capture long-term mate choice processes^83,84^. Specifically, Li and colleagues^84^ argue that speed-date designs might attract people that are not necessarily considering their interaction partners as long-term mates. Thus, they posit that the unique effects of short-term and long-term mate choice cannot be disentangled in speed-date designs, and that it is unclear whether such designs more closely resemble short-term or long-term mate choice contexts. However, it should be noted that almost all of our participants (except for 2) reported that they were interested in pursuing a long-term relationship and, in line with other speed-date events^12,17^, still seemed to value physical attractiveness, although this is often specifically mentioned as a criterium for short-term mates^84^. Furthermore, previous work has shown that long-term partner ratings and physical attractiveness ratings highly correlate^11,85,86^. In addition, it remains to be established whether there are specific contexts that emphasize long-term over short-term mate-choice considerations. In fact, a large-scale study showed no evidence that different initial meeting contexts (e.g., bars, church, online) influence divorce rates^87^. In a speed-date context, it has been shown that first impressions, which are asserted by some to reflect short-term mate choice processes, still predict long-term romantic interest^41^. In conclusion, our findings should not be interpreted as the product of uniquely long- or short-term mate choice processes. Instead, our findings would be best interpreted in the context of a close-relationships tradition^88^ that considers short-term and long-term contexts as closely related.

Finally, we attempted to disentangle the effects of attractiveness and date outcome on immediate and voluntary attention by means of Bayesian model comparisons (PSIS-LOO-CV^72^). For immediate attention, these comparisons suggest that pre-date ratings of attractiveness are more predictive of reaction times than date outcome or post-date attractiveness, even though the differences were not robust. Thus, we cannot draw strict conclusions regarding the relative influence of attractiveness and date outcome on immediate attention. For voluntary attention, on the other hand, we found robust evidence in favor of the model that includes pre-date attractiveness ratings over the models that include date outcome and post-date attractiveness rating, respectively. This suggests that voluntary attention is specifically driven by physical attractiveness ratings, which is in line with previous work^6,34,46^. Consequently, the robust effect of date outcome on voluntary attention might have been the result of strong intercorrelation between attractiveness ratings and date outcome, as has been reported in many studies^11,17^. To address this limitation, we suggest that future studies could employ a pre-post-design, where participants engage in attention tasks before and after a speed-date session to study specifically how the experiences gained during the speed-dates alter attentional processes.

Here, we investigated how attractiveness and date outcome were associated with immediate and voluntary attention in non-committed young adults. In line with previous studies, we found substantial inter-individual differences in attractiveness preferences. Furthermore, we found that immediate attention was modulated by attractiveness for men, but not for women, while no consistent relationship between immediate attention and date outcome was found. With regard to voluntary attention, we found that both men and women looked longer at pictures of people that they found attractive and that they wanted to date again. However, attractiveness ratings were more predictive of voluntary attention than date outcome. Our results therefore suggest that especially voluntary attention can provide information about individual preferences and possibly also mate choice of people who are motivated to find a partner.

## Supplementary Information

Supplementary Information.

## Data Availability

The datasets and materials generated and/or analysed during the current study are available via Dataverse: 10.34894/AWSZSB.
